# CTHRC1 Is a Prognostic Biomarker and Correlated With Immune Infiltrates in Kidney Renal Papillary Cell Carcinoma and Kidney Renal Clear Cell Carcinoma

**DOI:** 10.3389/fonc.2020.570819

**Published:** 2021-02-08

**Authors:** Fenfang Zhou, Dexin Shen, Yaoyi Xiong, Songtao Cheng, Huimin Xu, Gang Wang, Kaiyu Qian, Lingao Ju, Xinhua Zhang

**Affiliations:** ^1^ Department of Urology, Zhongnan Hospital of Wuhan University, Wuhan, China; ^2^ Department of Biological Repositories, Zhongnan Hospital of Wuhan University, Wuhan, China; ^3^ Human Genetics Resource Preservation Center of Hubei Province, Wuhan, China; ^4^ Laboratory of Precision Medicine, Zhongnan Hospital of Wuhan University, Wuhan, China

**Keywords:** collagen triple helix repeat containing 1 (CTHRC1), kidney renal clear cell carcinoma, kidney renal papillary cell carcinoma, prognosis, immune infiltration

## Abstract

Kidney renal clear cell carcinoma (KIRC) and kidney renal papillary cell carcinoma (KIRP) are the most common RCC types. RCC has high immune infiltration levels, and immunotherapy is currently one of the most promising treatments for RCC. Collagen triple helix repeat containing 1 (CTHRC1) is an extracellular matrix protein that regulates tumor invasion and modulates the tumor microenvironment. However, the association of *CTHRC1* with the prognosis and tumor-infiltrating lymphocytes of KIRP and KIRC has not been reported. We examined the *CTHRC1* expression differences in multiple tumor tissues and normal tissues *via* exploring TIMER, Oncomine, and UALCAN databases. Then, we searched the Kaplan-Meier plotter database to evaluate the correlation of *CTHRC1* mRNA level with clinical outcomes. Subsequently, the TIMER platform and TISIDB website were chosen to assess the correlation of *CTHRC1* with tumor immune cell infiltration level. We further explored the causes of aberrant *CTHRC1* expression in tumorigenesis. We found that *CTHRC1* level was significantly elevated in KIRP and KIRC tissues relative to normal tissues. *CTHRC1* expression associates with tumor stage, histology, lymph node metastasis, and poor clinical prognosis in KIRP. The *CTHRC1* level correlates to tumor grade, stage, nodal metastasis, and worse survival prognosis. Additionally, *CTHRC1* is positively related to different tumor-infiltrating immune cells in KIRP and KIRC. Moreover, *CTHRC1* was closely correlated with the gene markers of diverse immune cells. Also, high *CTHRC1* expression predicted a worse prognosis in KIRP and KIRC based on immune cells. Copy number variations (CNV) and DNA methylation might contribute to the abnormal upregulation of *CTHRC1* in KIRP and KIRC. In conclusion, *CTHRC1* can serve as a biomarker to predict the prognosis and immune infiltration in KIRP and KIRC.

## Introduction

Kidney cancer is among the top ten causes of cancer-related deaths. There are various subtypes of kidney cancer based on mixed histology, clinical course, and gene course. Renal cell carcinoma (RCC) is the most important type of kidney cancer (1). Besides, kidney renal clear cell carcinoma (KIRC) and kidney renal papillary cell carcinoma (KIRP) account for almost 95% of all renal cell cancers (2). The diagnosis, examination, surgery, and drug therapy of RCC have been advanced. However, its clinical outcome remains unsatisfactory (3, 4). RCC is a heterogeneous tumor that requires useful molecular markers suitable for personalized therapy (5, 6).

During cancer tumorigenesis and progression, tumor cells are affected by the tumor-infiltrating immune cells (7, 8). The immune invasion of the tumor is closely associated with the clinical prognosis of RCC. Precious studies indicated that tumor-infiltrating macrophages, regulatory T Cells (Treg cells), and CD8^+^ T cells influence RCC treatment outcomes (9–11). Besides, M1 macrophages are associated with better prognosis, while M2 macrophages predict poor outcome in KIRP. Immunoregulatory molecules CTLA-4 and LAG-3 associate with a poor prognosis in KIRC, while IDO1 and PD-L2 correlate with a poor prognosis in KIRP (12). These findings demonstrate that tumor infiltration of immune cells may be a useful drug target that improving clinical outcomes.

Tumor microenvironment comprises infiltrating immune cells, stromal cells, extracellular matrix, and tumor cells. Several studies have reported that tumor-infiltrating lymphocytes have different vital roles in tumor development. For example, tumor-associated macrophages (TAM) promotes cancer metastasis (13). CTHRC1 is a 30 kDa secreted protein, which is highly expressed in cartilage, developing bones, and myofibroblasts during skin wound healing and solid tumors (14, 15). Previous studies indicate that CTHRC1 promotes tumor cell progression *via* influencing specific pathways in various cancer types. CTHRC1 is elevated in cervical carcinoma and promotes metastasis through the Wnt/PCP pathway. In contrast, CTHRC1 modulates aggressiveness *via* GSK-3β/β-catenin pathway in human non-small cell lung cancer (16). Therefore, CTHRC1 is suggested to play an essential role in cancer progression. Current studies have found CTHRC1 function in modulating the tumor microenvironment *via* the E6/E7-p53-POU2F1 axis or focal adhesion kinase signal pathway (17, 18). In endometrial cancer, CTHRC1 promotes M2-like macrophage recruitment and myometrial invasion *via* the integrin-Akt signaling pathway (19). Thus, CTHRC1 has multifaceted functions in the tumor microenvironment. However, the underlying mechanisms of CTHRC1 in KIRP and KIRC progression and tumor-infiltrating lymphocytes remains unclear.

In this study, we used Oncomine, TIMER, UALCAN datasets, and Kaplan–Meier plotter web to analyze *CTHRC1* expression and its association with the prognosis. Furthermore, we used the TIMER web resource and TISIDB database to analyze the correlation between *CTHRC1* and tumor-infiltrated immune cells in the tumor microenvironment. Besides, we further explored the molecular mechanisms of CTHRC1 dysregulation, such as analysis of the CNV, DNA methylation, and somatic cell mutations. Our findings underline the vital role of CTHRC1 in KIRP and KIRC prognosis. Also, we provide an underlying mechanism of *CTHRC1* expression in potentially regulating the infiltration of immune cells, partly affecting the prognosis of KIRP and KIRC.

## Materials and Methods

### Oncomine Database Analysis

Oncomine database (https://www.oncomine.org/resource/main.html) integrates literature and databases of tumor microarray results and is mainly used for gene expression analysis, co-expression analysis, enrichment analysis, interaction networks (20). We used the Oncomine database to analyze *CTHRC1* expression in various cancer types.

### TIMER Database Analysis

TIMER web server (https://cistrome.shinyapps.io/timer/) is a website for comprehensive analysis of gene expression and tumor-infiltrating immune cells of diverse cancer types. This web assesses the abundances of six tumor-infiltrating cells (B cells, CD4^+^ T cells, CD8^+^ T cells, neutrophils, macrophages, and dendritic cells), using the TIMER algorithm (21). TIMER website also enables the user to explore gene expression in tumor tissues and normal tissues in multiple cancers. We used the TIMER website to analyze the differential expression of CTHRC1 in tumor and normal tissues in various cancers. We evaluated the correlation of CTHRC1 with 6 tumor immune infiltrating cells and molecular markers of 16 immune cells. We also used this web to explore the relationship between immune infiltrating cells and gene expression that affects clinical prognosis in KIRP and KIRC. The levels of gene expression were expressed as log2 RSEM.

### UALCAN Database Analysis

UALCAN database (http://ualcan.path.uab.edu/index.html) is available for online analysis of differential gene expression in cancer and normal tissue from the TCGA RNA sequencing data and clinical data of 31 malignancies (22). Besides, this website provides survival prognosis data based on gene expression differences in 31 cancer types. This study used the UALCAN database to validate the analysis results of the Oncomine database, and furtherly determined the correlation between *CTHRC1* gene expression and clinical features. Differences at p<0.05 were considered statistically significant.

### Kaplan-Meier Plotter Database Analysis

Kaplan-Meier plotter (http://kmplot.com/analysis/) (23) is an open, intuitive portal tool for prognostic analysis. It contains 54,675 genes survival data from 10,461 cancer samples. Kaplan-Meier plotter database was used to assess the relationship between clinic outcomes and *CTHRC1* expression in different cancers. We performed a prognostic analysis based on *CTHRC1* expression levels in relevant immune cell subgroups using this web. We calculated hazard ratios (HRs) of 95% confidence intervals (CIs) and the log-rank p-value.

### TISIDB

TISIDB database (http://cis.Hku.hk/TISIDB/) is a portal for analyzing tumor and immune cell interactions that integrates multiple heterogeneous data types (24). We analyze the correlation between *CTHRC1* expression and tumor-infiltrating lymphocytes *via* this platform.

### UCSC Xena

UCSC Xena database (http://xena.ucsc.edu/) is a genome-related database, which brings approximately 200 public databases together, including TCGA, ICGC, TARGET, GTEx, CCL, etc. (25). The database is available to examine copy number and methylation, somatic mutation, gene expression, protein expression. This web also provides clinical information such as patient treatment and survival.

### DiseaseMeth Version 2.0

The Human Disease Methylation Database (http://bioinfo.hrbmu.edu.cn/diseasemeth/) is an interactive database that provides annotation and analysis of abnormal DNA methylation in human diseases, especially cancers, which includes 32701 samples, 88 diseases, 679602 disease-gene associations (26).

### Statistical Analysis

The *CTHRC1* expression was analyzed *via* the Oncomine, TIMER, and UALCAN database. Survival curves were generated using the Kaplan-Meier plotter database and R project using “survival” packages. We used Spearman’s correlation analysis to evaluate the correlation of gene expression in the TIMER. p<0.05 were considered statistically significant.

## Results

### The *Collagen Triple Helix Repeat Containing 1* mRNA Expression in Different Cancers

**Figure 1:**
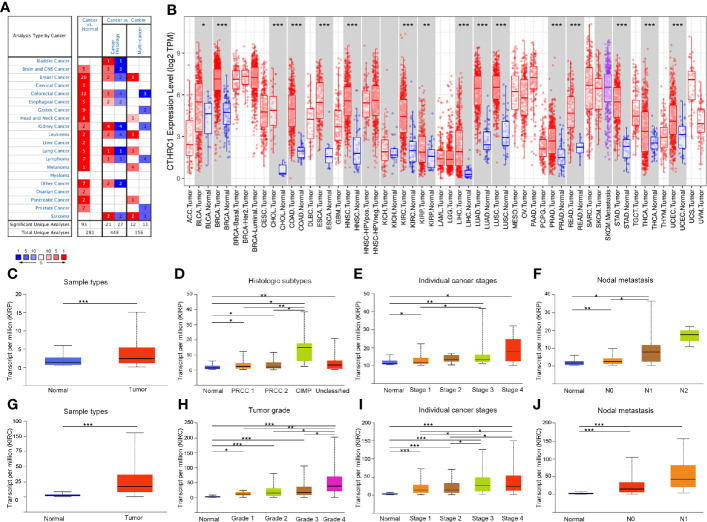
The expression of *CTHRC1* in different cancers and its relationship with individual clinical parameters of KIRP and KIRC. **(A)** *CTHRC1* level in different cancers tissues compared to normal tissues in the Oncomine database. **(B)** *CTHRC1* expression of different tumor types in the TIMER database. **(C)** *CTHRC1* expression difference in KIRP samples. **(D–F)** *CTHRC1* mRNA expressions were remarkably correlated with KIRP patients’ individual cancer histologic subtypes **(D)**, stages **(E)**, nodal metastasis **(F)**. **(G)** Differential expression of *CTHRC1* in KIRC tissues. **(H–J)** *CTHRC1* level were significantly associated with KIRC patients’ individual cancer grade **(H)**, stages **(I)**, nodal metastasis **(J)**. N0: Metastases in 1 to 3 axillary lymph nodes, N1: Metastases in 1 to 3 axillary lymph nodes, N2: Metastases in 4 to 9 axillary lymph nodes. *p<0.05, **p<0.01, ***p<0.001.

We analyzed the mRNA expression of *CTHRC1* using the Oncomine database. The results showed that *CTHRC1* was significantly high in various cancer tissues, compared to normal tissues (**Figure 1A**). Then, the mRNA level in the TIMER database was determined. We found that *CTHRC1* mRNA expression was significantly high in most human tumors, especially in KIRP and KIRC, compared with the corresponding normal tissues (**Figure 1B**). These results showed that *CTHRC1* was highly expressed in various cancers. Besides, we used the UALCAN database to validate the findings in Oncomine and TIMER web and reported higher expression of *CTHRC1* in KIRP and KIRC tissues than in normal tissues (**Figures 1C, G**). Notably, *CTHRC1* expression was associated with tumor histology, stage, lymph node metastasis in KIRP (**Figures 1D–F**). Meanwhile, the high *CTHRC1* level in KIRC was related to lymph node metastasis high grade and stage (**Figures 1H–J**).

### Prognostic Significance of *Collagen Triple Helix Repeat Containing 1* Expression in Human Cancers

**Figure 2:**
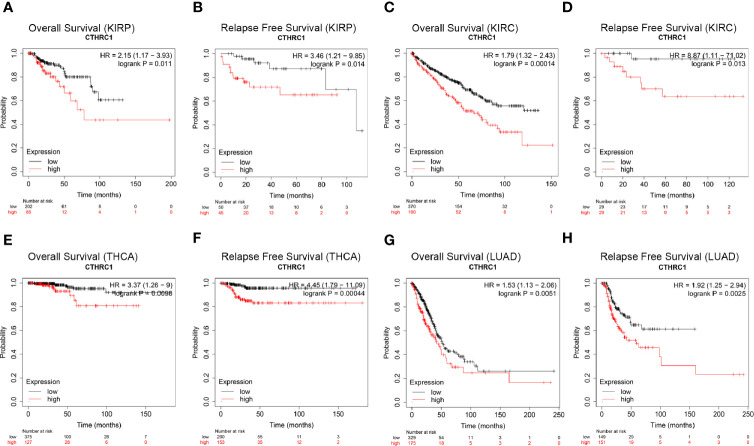
Comparison of Kaplan-Meier survival curves of *CTHRC1* high and low expression in different cancers. **(A, B)** High *CTHRC1* expression had poor OS and RFS in KIRP (n=288). **(C, D)** Upregulated *CTHRC1* expression had worse OS and RFS in KIRC (n=530). **(E, F)** Difference in survival among high and low *CTHRC1* levels in THCA (n=502). **(G, H)** Survival differences of *CTHRC1* expression in LUAD (n=513).

**Figure 3:**
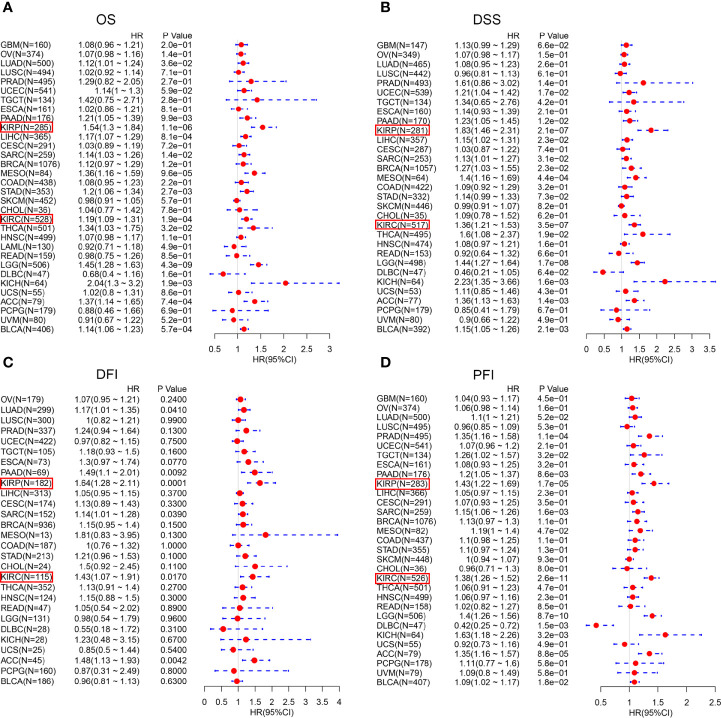
Forest plot of the prognostic values in various cancer subgroups of *CTHRC1*. **(A–D)** Prognostic HR of *CTHRC1* in different cancers for OS **(A)**, DSS **(B)**, DFI **(C)**, PFI **(D)**.

We investigated the Kaplan-Meier plotter database for the prognostic significance of *CTHRC1* expression in human cancers. High levels of *CTHRC1* predicted poor prognostic in KIRP (**Figures 2A, B**), KIRC (**Figures 2C, D**), THCA (**Figures 2E, F**), and LUAD (**Figures 2G, H**). As Kaplan-Meier plotter analyzes only OS and RFS value, we assessed the multiple clinical prognostic value of *CTHRC1* in a variety of cancers by R project using “survival” packages. Forest plot showed *CTHRC1* as a risk factor of different prognosis in KIRP and KIRC (**Figure 3**). Besides, we generated the Kaplan-Meier plot, which showed that high expression of *CTHRC1* had a poor prognosis in KIRP (**Supplementary Figures S1A–D**) and KIRC (**Supplementary Figures S1E–H**). These findings indicated that *CTHRC1* is a hazard for predicting worse prognostic in KIRP and KIRC.

### Correlation of *Collagen Triple Helix Repeat Containing 1* Expression With Clinical Characteristics of Kidney Renal Papillary Cell Carcinoma and Kidney Renal Clear Cell Carcinoma Patients

Then, we investigated the association of *CTHRC1* expression with different clinical characteristics of KIRP and KIRC using the Kaplan-Meier Plotter database (**Table 1**). High *CTHRC1* level was associated with poorer OS and RFS in females (OS: HR=7.87, p=0.00021; RFS: HR=8.01, p=0.0022) and stage 3 (OS: HR=4.87, p=0.0028; RFS: HR=9.2, p=0.00021) in KIRP. Similarly, upregulated levels of *CTHRC1* was correlated with worse prognostic outcomes in males (OS: HR=1.71, p=0.0143; RFS: HR=5.44, p=0.0025), and stage 2 (OS: HR=13.51, p=0.0021), stage 3 (RFS: HR=4.65, p=0.0457), stage 4 (OS: HR=1.72, p=0.0345) in KIRC. These results illustrate that the prognostic value of the *CTHRC1* mRNA level, following their clinical characteristics, particularly in the advanced stage of KIRP and KIRC patients.

**Table 1:** Correlation of *CTHRC1* mRNA expression and prognosis in KIRP and KIRC with different clinicopathological factors by Kaplan-Meier plotter.

Clinicopathological factors	KIRP				KIRC			
	OS		RFS		OS		RFS	
	HR	p	HR	p	HR	p	HR	p
SEX								
Female	7.87(2.16–28.65)	0.00021	8.01(1.68–38.26)	0.0022	3.58(2.11–6.08)	5.00E-07	1.71(0.24–12.18)	0.587
Male	5.61(1.34–23.50)	0.0078	2.55(0.93–7.00)	0.06	1.71(1.11–2.65)	0.0143	5.44(1.59–18.67)	0.0025
Stage								
1	3.15(0.92–10.75)	0.054	1.8(0.58–5.587)	0.3	1.7(0.92–3.11)	0.085	2.97(0.31–28.72)	0.323
2	0(0-lnf)	0.36	–	–	13.51(1.69–107.66)	0.0021	–	–
3	4.87(1.55–15.37)	0.0028	9.2(2.25–37.59)	0.00021	1.51(0.75–3.02)	0.2451	4.65(0.89–24.47)	0.0457
4	–	–	–	–	1.72(1.13–2.87)	0.0345	–	–

### *Collagen Triple Helix Repeat Containing 1* Expression is Correlated With Immune Infiltration in Kidney Renal Papillary Cell Carcinoma and Kidney Renal Clear Cell Carcinoma

**Figure 4:**
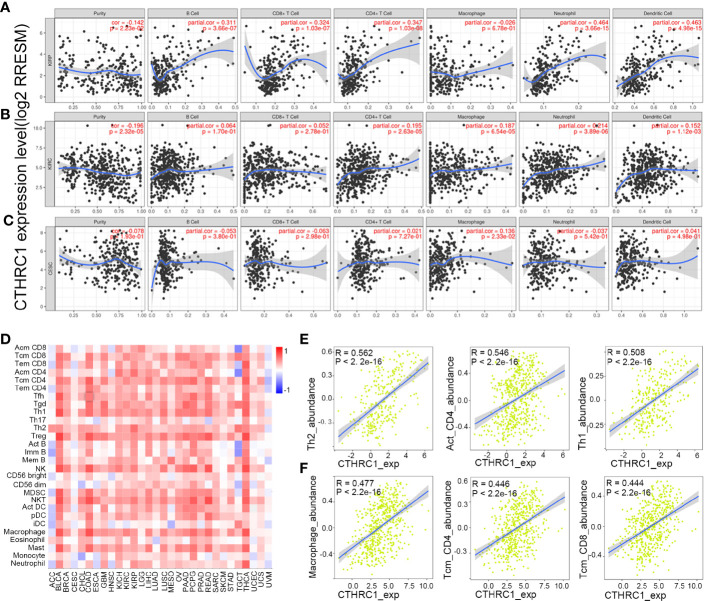
Correlation analysis of *CTHRC1* level and immune cells infiltration levels across human cancers using the TIMER database and TISIDB database. **(A)** *CTHRC1* expression in KIRP tissues positive correlates with tumor immune infiltration levels of B cells, CD8^+^ T cells, CD4^+^ T cells, neutrophils, and dendritic cells. **(B)** *CTHRC1* level correlates with tumor immune infiltration in KIRC. **(C)** *CTHRC1* level weakly correlated with immune cells infiltration of CESC. **(D)** Relations between expression of *CTHRC1* and 28 types of TILs across human heterogeneous cancers. **(E)** Top 3 TILs were displaying the greatest Spearman’s correlation with *CTHRC1* expression in KIRP. **(F)** *CTHRC1* significantly correlated with abundance of top 3 TILs in KIRC.

Tumor-infiltrating lymphocytes can independently be used to predict sentinel lymph node status and prognosis in cancers (27, 28). Therefore, we used TIMER to analyze the correlation of *CTHRC1* level with immune infiltration levels in various cancer types. The results showed that *CTHRC1* expression is significantly positively correlated with B cells (r=0.311, p=3.66e-07), CD4^+^ T cells (r=0.347, p=1.03e-08), CD8^+^ T cells (r=0.324, p=1.03e-07), dendritic cells (r=0.463, p=4.98e-15) and neutrophils (r=0.464, p=3.66e-15) in KIRP (**Figure 4A**). Besides, the *CTHRC1* level showed a positive correlation with infiltrating levels of CD4^+^ T cells (r=0.195, p=2.63e-05), neutrophils (r=0.214, p=3.89e-06), macrophage (r=0.187, p=6.54e-04), dendritic cell (r=0.152, p=1.12e-03) in KIRC (**Figure 4B**). However, *CTHRC1* was not correlated with B cells (r=−0.053, p=3.80e-01), CD4^+^ T cells (r=0.021, p=7.27e-01), CD8^+^ T cells (r=−0.063, p=2.98e-01), dendritic cells (r=0.041, p=4.98e-01), and neutrophils (r=−0.037, p=5.42e-01) in cervical and endocervical cancers (**Figure 4C**). In addition, we examined prognostic value of *CTHRC1* level and tumor infiltrating immune cells in KIRP and KIRC, using Cox proportional hazard model by TIMER. The result states that B cells (p=0.039), CD8^+^ T cells (p=0.001), dendritic cells (p=0.018), *CTHRC1* expression (p<0.001) were significantly correlated with clinical prognosis in KIRP (**Table 2**). Besides, there is a strong correlation between Macrophage (p=0.020), *CTHRC1* expression (p<0.001) and clinical outcome of KIRC (**Table 2**). Then, we used TISIDB database to furtherly explore the relationship between *CTHRC1* level and 28 tumor immune infiltrating cell subtypes. These results showed that *CTHRC1* is associated with twenty-seven immune cell subtypes in KIRP (**Figure 4D**, **Table 3**). Especially, effector memory CD8^+^ T cell (r=0.414, p=1.81E-13), activated CD4 T cell (r=0.546, p<2.2e-16), Type 1 T helper cell (r=0.454, p<2.2e-16), Type 2 T helper cell (r=0.562, p<2.2e-16), regulatory T cell (r=0.435, p<2.2e-16), activated B cell (r=0.409, p=4.11e-13), natural killer cell (r=0.493, p<2.2e-16), natural killer T cell (r=0.508, p<2.2e-16), neutrophil (r=0.422, p=3.40E-14) and *CTHRC1* are moderately correlated (**Figure 4E**). In addition, *CTHRC1* showed a positive correlation with 24 immune cell (**Figure 4D**, **Table 3**). Notably, central memory CD8^+^ T cell (r=0.444, p<2.2e-16), central memory CD4^+^ T cell (r=0.446, p<2.2e-16), gamma delta T cell (r=0.43, p<2.2e-16), macrophage (r=0.477, p<2.2e-16) displayed a moderate correlation with *CTHRC1* expression (**Figure 4F**). These results strongly implicate that *CTHRC1* could serve as a major tumor immune infiltration regulator in KIRP and KIRC.

**Table 2:** The cox proportional hazard model of CTHRC1 and six tumor-infiltrating immune cells in KIRP and KIRC (TIMER).

	KIRP					KIRC				
	coef	HR	95%CI_l	95%CI_u	p.value	coef	HR	95%CI_l	95%CI_u	p.value
B cell	5.238	188.256	1.311	27041.674	0.039	−0.496	0.609	0.027	13.546	0.754
CD8 T cell	9.748	17125.920	44.033	6660832.100	0.001	−1.126	0.324	0.065	1.629	0.171
CD4 T cell	−1.312	0.269	0.000	725.972	0.745	−0.615	0.541	0.037	7.928	0.654
Macrophage	−3.662	0.026	0.000	4.235	0.160	−2.793	0.061	0.006	0.644	0.020
Neutrophil	−9.450	0.000	0.000	63.944	0.173	2.477	11.910	0.209	679.922	0.230
Dendritic	−5.194	0.000	0.000	0.416	0.018	0.561	1.753	0.289	10.623	0.542
CTHRC1	0.649	1.913	1.435	2.551	0.000	0.205	1.228	1.110	1.359	0.000

**Table 3:** The correlation between CTHRC1 expression and tumor lymphocyte infiltration in human cancer (TISIDB).

	KIRP		KIRC	
	r	p	r	p
Activated CD8 T cell (Act _CD8)	0.358	4.39E-10	0.184	1.86E-05
Central memory CD8 T cell (Tcm _CD8)	0.355	5.96E-10	0.444	<2.2e-16
Effector memory CD8 T cell (Tem _CD8)	0.414	1.81E-13	0.19	1.01E-05
Activated CD4 T cell (Act _CD4)	0.546	<2.2e-16	0.328	1.00E-14
Central memory CD4T cell (Tcm _CD4)	0.340	3.45E-09	0.446	<2.2e-16
Effector memory CD4 T cell (Tem _CD4)	0.328	1.24E-08	0.259	1.47E-09
T follicular helper cell(Tfh)	0.210	9.73E-13	0.311	2.56E-13
Gamma delta T cell (Tgd)	0.336	<2.2e-16	0.430	<2.2e-16
Type 1 T helper cell (Th1)	0.454	<2.2e-16	0.360	3.54E-08
Type 17 T helper cell (Th17)	0.157	7.20E-03	0.072	9.55E-01
Type 2 T helper cell (Th2)	0.562	<2.2e-16	0.337	1.48E-15
Regulatory T cell (Treg)	0.435	<2.2e-16	0.349	1.15E-16
Activated B cell (Act _B)	0.409	4,11e-13	0.184	2.03E-05
Immature B cell (Imm _B)	0.387	1.09E-10	0.118	6.19E-03
Memory B cell (Mem _B)	0.353	7.88-10	0.303	1.09E-12
natural killer cell (NK)	0.493	<2.2e-16	0.324	2.31E-14
CD56bright natural killer cell (CD56bright)	0.206	4.23E-03	0.205	1.86E-06
CD56dim natural killer cell (CD56dim)	0.231	7.26E-05	0.142	1.02E-03
Myeloid derived suppressor cell (MDSC)	0.351	9.96-10	0.321	4.00E-14
Natural killer T cell (NKT)	0.508	<2.2e-16	0.380	<2.2e-16
Activated dendtritic cell (Act _DC)	0.357	4.49E-10	0.163	1.63E-03
Plasmacytoid dendtritic cell (pDC)	0.386	1.27E-11	0.285	2.38E-11
Immature dendtritic cell (iDC)	0.048	4.17E-01	-0.101	2.00E-02
Macrophage (Macrophage)	0.393	5.09E-12	0.477	<2.2e-16
Eosinophi (Eosinophil)	0.361	2.87E-10	0.053	2.24E-01
Mast (Mast)	0.344	2.15E-09	0.304	9.37E-13
Monocyte(Monocyte)	0.237	4.78E-05	0.213	7.37E-13
Neutrophil (Neutrophil)	0.422	3.40E-14	0.035	4.13E-01

### *Collagen Triple Helix Repeat Containing 1* Expressions Were Correlated With Immune Cell Type Markers

We assessed the correlation between *CTHRC1* expression and tumor-infiltrating immune cell gene marker levels in KIRP, KIRC, and CESC tissues by exploring the TIMER database. Our results showed that the *CTHRC1* level in KIRP tissues was strongly associated with immune markers of B cells, monocytes, and dendritic neutrophils, DCs, CD8^+^ T cells, T helper, Tregs, and T exhaustion cells. Moreover, *CTHRC1* in KIRC positively correlates with the marker genes of B cells, macrophages, and neutrophils. However, there were only ten significant markers associated with *CTHRC1* in CESC.

Notably, we found that the *CTHRC1* level was significantly correlated with various subtypes of T cells marker levels, including CD8^+^ T markers, CD8A, CD8B, cell T (general) markers, CD3D, CD3E, CD2, exhausted T cell marker, GZMB, LAG-3, PD-1, Th2 markers, GATA3, Th17 markers, STAT3, Treg markers, FOXP3, CCR8, TGF-β, Tfh marker, BCL6, and neutrophils markers, ITGAM, CCR7, DC markers, CD1C, NRP1, B cells markers, CD79A, CD19 in KIRP (**Table 4**). A significant correlation of *CTHRC1* level with the marker genes expression in different subsets of macrophage. M2 macrophage markers MS4A4A, VSIG4, CD163, monocyte markers, CSF1R, CD86, TAM markers, CD68, B cell markers, CD19, CD79A, was reported (**Table 4**). Furthermore, the expression of *CTHRC1* was not markedly related to marker genes for CD8^+^ T cells, NK cells, Th2, and Th17 cells in KIRC. These findings reveal that CTHRC1 is involved in the regulation of tumor immune infiltration in KIRP and KIRC.

**Table 4:** Correlation analysis between CTHRC1 and relate genes and markers of immune cells in TIMER.

Description	Gene markers	KIRP				KIRC				CESC			
		None		Purity		None		Purity		None		Purity	
		cor	p	cor	p	cor	p	cor	p	cor	p	cor	p
B cell	CD19	0.375	***	0.392	***	0.295	***	0.301	***	−0.099	0.083	−0.106	0.078
	CD79A	0.489	***	0.489	***	0.246	***	0.260	***	−0.064	0.267	−0.061	0.311
CD8^+^ T cell	CD8A	0.403	***	0.425	***	0.082	0.059	0.091	0.050	−0.062	0.276	−0.079	0.188
	CD8B	0.355	***	0.390	***	0.054	0.214	0.065	0.161	−0.044	0.441	−0.062	0.307
Dendritic cell	ITGAX	0.221	**	0.213	**	0.068	0.114	0.051	0.270	0.046	0.419	0.036	0.547
	NRP1	0.330	***	0.328	***	0.220	***	0.220	***	0.293	***	0.298	***
	CD1C	0.430	***	0.453	***	0.045	0.302	0.032	0.491	0.168	*	0.170	*
	HLA-DPA1	0.295	***	0.307	***	0.099	0.023	0.113	0.015	−0.085	0.136	−0.104	0.083
	HLA-DRA	0.302	***	0.313	***	0.126	*	0.141	*	−0.094	0.102	−0.116	0.054
	HLA-DQB1	0.235	***	0.275	***	0.019	0.660	0.022	0.638	−0.082	0.153	−0.112	0.062
	HLA-DPB1	0.285	***	0.307	***	0.106	0.015	0.119	0.010	−0.102	0.074	−0.120	0.046
M1 Macrophage	PTGS2	0.397	***	0.417	***	0.322	***	0.326	***	0.201	**	0.187	*
	IRF5	−0.124	0.030	−0.153	0.014	−0.112	*	−0.125	*	−0.023	0.688	−0.008	0.901
	NOS2	0.173	*	0.205	**	−0.005	0.900	−0.017	0.712	0.012	0.832	0.012	0.844
M2 Macrophage	MS4A4A	0.304	***	0.298	***	0.307	***	0.310	***	0.145	0.011	0.120	0.045
	VSIG4	0.337	***	0.342	***	0.352	***	0.350	***	0.128	0.025	0.118	0.049
	CD163	0.317	***	0.323	***	0.289	***	0.293	***	0.121	0.034	0.114	0.058
Monocyte	CSF1R	0.353	***	0.374	***	0.279	***	0.276	***	0.149	*	0.141	0.019
	CD86	0.351	***	0.363	***	0.256	***	0.263	***	0.132	0.021	0.101	0.093
Natural killer cell	KIR2DS4	0.236	***	0.300	***	0.044	0.312	0.072	0.124	−0.068	0.236	−0.068	0.260
	KIR3DL3	0.128	0.030	0.126	0.044	0.014	0.743	0.031	0.505	−0.111	0.052	−0.148	0.013
	KIR3DL2	0.309	***	0.345	***	0.079	0.069	0.116	0.012	−0.110	0.054	−0.127	0.035
	KIR3DL1	0.330	***	0.360	***	0.047	0.276	0.072	0.121	0.008	0.885	−0.010	0.866
	KIR2DL4	0.332	***	0.361	***	0.082	0.059	0.099	0.033	−0.141	0.013	−0.176	*
	KIR2DL3	0.293	***	0.334	***	0.047	0.280	0.072	0.122	−0.010	0.865	−0.047	0.436
	KIR2DL1	0.263	***	0.286	***	0.043	0.318	0.073	0.115	−0.076	0.187	−0.090	0.133
Neutrophils	CCR7	0.371	***	0.372	***	0.239	***	0.269	***	0.009	0.875	0.020	0.743
	ITGAM	0.404	***	0.424	***	0.195	***	0.180	**	0.064	0.261	0.053	0.382
	CEACAM8	0.049	0.410	0.061	0.331	−0.127	*	−0.112	0.016	−0.047	0.415	−0.024	0.693
T cell (general)	CD3D	0.392	***	0.413	***	0.131	*	0.136	*	−0.126	0.028	−0.150	0.012
	CD3E	0.411	***	0.446	***	0.140	0.001	0.146	*	−0.063	0.270	−0.079	0.187
	CD2	0.395	***	0.425	***	0.155	**	0.158	**	−0.061	0.286	−0.081	0.179
T cell exhaustion	CTLA4	0.272	***	0.290	***	0.151	**	0.153	**	−0.009	0.881	−0.033	0.582
	LAG3	0.411	***	0.424	***	0.103	0.018	0.105	0.024	−0.072	0.209	−0.102	0.090
	HAVCR2	0.013	0.832	0.004	0.951	−0.175	***	−0.175	**	0.069	0.228	0.047	0.434
	GZMB	0.446	***	0.472	***	0.135	*	0.159	**	−0.107	0.062	−0.133	0.026
	PDCD1	0.353	***	0.374	***	0.071	0.101	0.084	0.070	−0.074	0.196	−0.091	0.129
TAM	CCL2	0.313	***	0.317	***	−0.036	0.404	−0.052	0.263	0.202	**	0.202	**
	IL10	0.286	***	0.292	***	0.195	***	0.199	***	0.180	*	0.183	*
	CD68	0.070	0.234	0.057	0.361	0.260	***	0.277	***	−0.033	0.562	−0.065	0.277
Tfh	BCL6	0.390	***	0.384	***	0.290	***	0.297	***	0.227	***	0.210	**
	IL21	0.125	0.033	0.131	0.035	0.150	**	0.160	**	−0.027	0.636	−0.037	0.539
Th1	TBX21	0.345	***	0.397	***	0.080	0.065	0.093	0.045	−0.055	0.341	−0.083	0.169
	STAT4	0.271	***	0.286	***	0.191	***	0.211	***	0.042	0.459	0.008	0.897
	STAT1	0.380	***	0.396	***	0.125	*	0.119	0.011	0.106	0.065	0.068	0.260
	IFNG	0.284	***	0.313	***	0.108	0.013	0.116	0.013	−0.024	0.676	−0.059	0.323
	IL13	0.099	0.091	0.086	0.166	−0.034	0.429	−0.015	0.752	0.093	0.105	0.106	0.077
Th2	GATA3	0.453	***	0.474	***	0.099	0.022	0.061	0.192	0.155	*	0.146	0.015
	STAT6	0.162	*	0.153	0.014	−0.114	*	−0.102	0.028	0.044	0.447	0.061	0.312
	STAT5A	0.218	**	0.242	***	0.109	0.011	0.101	0.030	−0.110	0.055	−0.109	0.069
Th17	STAT3	0.425	***	0.441	***	0.110	0.011	0.095	0.042	0.067	0.242	0.075	0.210
	IL17A	0.126	0.033	0.111	0.073	0.108	0.012	0.118	0.011	−0.142	0.013	−0.137	0.023
Treg	FOXP3	0.415	***	0.438	***	0.329	***	0.340	***	0.151	*	0.130	0.031
	CCR8	0.362	***	0.373	***	0.259	***	0.272	***	0.221	***	0.192	*
	STAT5B	0.166	*	0.170	*	−0.124	*	−0.120	0.010	0.206	**	0.196	*
	TGFB1	0.516	***	0.563	***	0.444	***	0.423	***	0.337	***	0.315	***

*p<0.01; **p<0.001; ***p<0.0001.

### Prognostic Potential of *Collagen Triple Helix Repeat Containing 1* Expressions in Different Tumors Based on Immune Cells

This study showed that the *CTHRC1* level was associated with the immune infiltration of KIRP and KIRC. Also, upregulated *CTHRC1* has a worse prognosis in KIRP and KIRC patients. Thus, we propose a hypothesis that CTHRC1 may affect the prognosis of KIRP and KIRC patients partly through immune infiltration.

**Figure 5:**
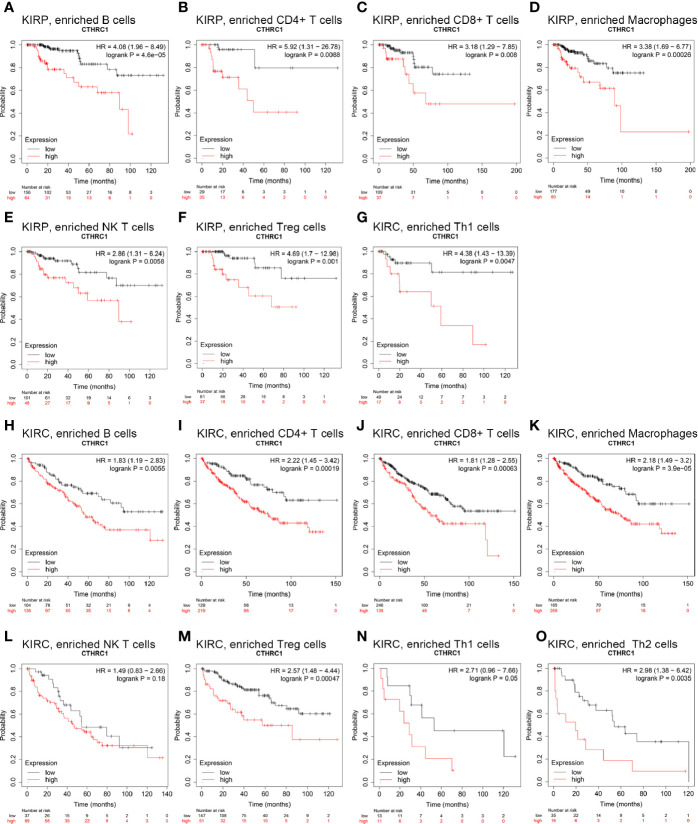
Comparison of Kaplan-Meier survival curves of the high and low expression of CTHRC1 in KIRP and KIRC based on immune cells subgroups. **(A–G)** High *CTHRC1* level enriched in B cells, CD4^+^ memory T cells, CD8^+^ T cells, macrophages, NK T cells, Treg T cells, Th1 cells had worse OS in KIRP. **(H–O)** Relationships between *CTHRC1* of enriched in diverse immune and OS in KIRC.

We perform Kaplan-Meier plotter analyses of *CTHRC1* expression in KIRP and KIRC following B cells, CD4+ memory T cells, CD8^+^ T cells, macrophages, NK T cells, Treg T cells, Th1 cells, Th2 cells. We found that high *CTHRC1* levels in KIRP in enriched B cells (p=0.00017), B cells (p=4.6e-05), CD4^+^ memory T cells (p=8.8e-03), CD8^+^ T cells (p=8e-03), macrophages (p=2.6e-04), natural killer T cells (p=5.8e-03), regulatory T cells (p=1e-03), type 1 T helper cells (p=4.7e-03) cohort had a worse prognosis (**Figures 5A–G**). Unfortunately, clinical samples of Th2 cells enriched in renal cancer are too few to analyze. Similarly, the high expression of *CTHRC1* in KIRC had poor prognosis in enriched B cells (p=5.5e-03), CD4^+^ memory T cells (p=1.9e-04), CD8^+^ T cells (p=6.3e-04), macrophages (p=3.9e-05), regulatory T cells (p=4.7e-04), type 2 T-helper cells (p=3.5e-03) (**Figures 5H–K, M, O**). However, there was no significant difference between high and low *CTHRC1* expression groups overall survival in enriched NK cells (p=0.18) and Th1 cells (p=0.05) (**Figures 5L, N**). The above analysis suggested that immune infiltration may, in part, affect high *CTHRC1* expression prognosis of KIRC and KIRP patients.

### Mutation, Copy Number Variation, and Methylation Analysis of *Collagen Triple Helix Repeat Containing 1*

**Figure 6:**
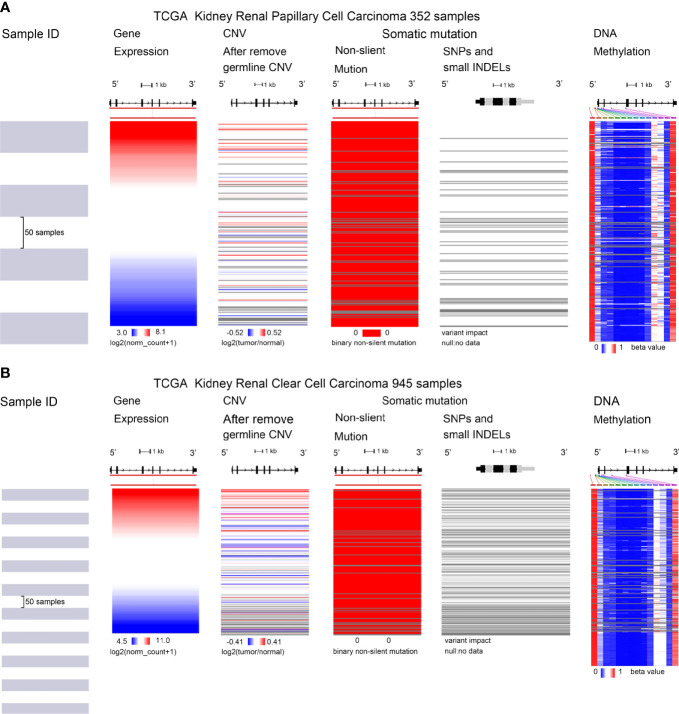
Mutation, CNV, and methylation analysis of CTHRC1 in KIRP and KIRC. **(A, B)** Heatmap showing the correlations between *CTHRC1* mRNA and somatic mutations, CNV, and methylation in KIRP **(A)** and KIRC **(B)** *via* UCSC Xena.

*CTHRC1* expression was significantly elevated in KIRP and KIRC. We assessed the cause of elevated *CTHRC1* levels. DNA methylation, gene mutation, CNV was critically involved in genetic and epigenetic regulation and were highly associated with the process of cancers. We verified the DNA methylation, gene mutation, CNV levels of the CTHRC1 in KIRP and KIRC *via* the UCSC Xena database. The heatmap indicates that the expression of *CTHRC1* mRNA was correlated with CNV and DNA methylation, but not with a somatic mutation in KIRP (**Figure 6A**) and KIRC (**Figure 6B**). The human disease methylation database was used to further validate the lower methylation level in KIRP (**Supplementary Figures S2A, B**) and KIRC (**Supplementary Figures S2C, D**), compared to normal tissues. Therefore, we suggested that CNV and DNA methylation might contribute to the elevated level of *CTHRC1* in KIRP and KIRC, respectively.

## Discussion

*CTHRC1* is an extracellular matrix protein that regulates tumor metastasis and the extracellular microenvironment. In this study, we analyzed *CTHRC1* expression, prognostic value, genetic variations, and correlation with tumor immune cell infiltration in KIRP and KIRC for the first time.

In this study, we found that *CTHRC1* expression was highly elevated in KIRP and KIRC, compared to normal tissues. Moreover, *CTHRC1* expression has associations with tumor histology, stage, lymph node metastasis in KIRP (**Figures 1D–F**). Meanwhile, the high *CTHRC1* level was related to lymph node metastasis, high grade, and stage (**Figures 1H–J**). These results suggest that *CTHRC1* plays an important role in the progression and metastasis of KIRP and KIRC. Our findings are consistent with previous researches. *CTHRC1* was elevated in some tumor tissues and associated with clinicopathological features, including late T stage, lymph nodal metastasis, and TNM staging (17, 29).

Results from survival analysis showed that high *CTHRC1* expression was associated with poor OS, DSS, DFI, and PFI (**Figure S1**) in KIRP and KIRC, consistent with previous findings *CTHRC1* affects tumor growth and invasion and leads to a poor prognosis (30, 31). Ni et al. (32) reported that *CTHRC1* promotes metastasis through an epithelial-mesenchymal transformation in colorectal cancer, resulting in a poor prognosis. Our results strongly indicate that *CTHRC1* can be used as a prognostic biomarker for KIRP and KIRC.

CTHRC1 was previously reported to regulate tumor microenvironment. Through correlation analysis, we reported that *CTHRC1* expression is associated with several immune infiltrating cells in KIRP and KIRC (**Figure 4**, **Table 3**). These results suggest that CTHRC1 is involved in the regulation of tumor immune cells. Lee et al (33). study revealed that CTHRC1 recruits Tie2-expressing monocytes into tumor tissues by activating ERK-dependent AP-1 to promote angiogenesis. Besides, the use of CTHRC1 antibodies reduced tumor burden and TEMs infiltration in tumor tissue in xenograft mouse models. Another study demonstrated that *CTHRC1* expression has a role in tumor-associated macrophages infiltration by upregulating fractalkine chemokine receptor (CX3CR1) expression (19). Our analysis had the effect of mutual authentication with the results of this researches.

We further analyzed the immunotype markers in KIRP and KIRC. After cell purity correction, *CTHRC1* was positively correlated with many immune cell makers in KIRP and KIRC (**Table 4**). The results further imply that *CTHRC1* is associated with immune infiltration in KIRP and KIRC. Besides, our results suggest that *CTHRC1* can potentially modulate Tregs and results in T cell exhaustion. Notably, increased *CTHRC1* level was positively associates with Treg and T cells exhaustion markers, such as FOXP3. FOXP3 is a valid target for identifying Treg in the tumor microenvironment and contributes significantly to Treg cells differentiation and mediated tumor immune escape (34). There was a significant correlation between *CTHRC1* level and several T helper cells (Th1, Th2, Tfh, and Th17) markers in KIRP. These connections may indicate the underlying mechanisms for *CTHRC1* regulation of T cell function in KIRP. Therefore, it was potentially related to the poor prognosis of KIRP and KIRC by recruiting and regulating immune cells.

Through the Kaplan Meier-Plotter database analysis, high expression levels of *CTHRC1* enriched in a variety of immune cells cohort of KIRP and KIRC had a worse prognosis (**Figure 5**). Tregs can suppress anti-tumor responses, leading to tumor immune escape (35). DC can promote tumor metastasis by increasing Treg cells and decreasing the cytotoxicity of CD8^+^ T cells (36). Myeloid origin suppressor cells (MDSC) contact with T cells, which rapidly depletes arginine from the microenvironment and leads to tumor-mediated immune escape (37). Previous studies also have proven that the proportion of macrophages, CD8^+^ T cells, Tregs, and MDSC in RCC patients correlates with poor prognosis (9–11, 38, 39). These results may explain that high expression of *CTHRC1* partly affects the prognosis of KIRP and KIRC patients through immune infiltration.

Genetic and epigenetic phenomena play an essential role in regulating gene expression (40). In this study, we found that *CTHRC1* expression was strongly correlated with DNA methylation and CNV and not with somatic mutations. DNA methylation is the most common epigenetic phenotype that usually acts as a transcriptional repressor and plays an essential role in tumor progression (41, 42). Besides, *CTHRC1* is reported to be upregulated by promoter demethylation in gastric cancer and down-regulated by hypermethylation in hepatocellular carcinoma (43). DNA copy number variation, including gene amplification, gain, loss, and deletion. And CNV influences the gene expression in carcinogenesis (44). Wang et al. (45) presented the evidence that 5-aza-2’-deoxycytidine (the demethylating agent) can restore *CTHRC1* expression, and TGF-β1 led to an increase in levels of *CTHRC1* mRNA and protein. Therefore, DNA hypomethylation and CNV may be a cause for *CTHRC1* upregulated in KIRP and KIRC.

In conclusion, the upregulated *CTHRC1* is strongly associated with clinicopathological features, poor prognosis, and immune cell infiltration. DNA methylation and copy number variation may attribute to *CTHRC1* upregulated. Furthermore, our study provides a new mechanism that CTHRC1 may affect the prognosis of KIRP and KIRC through tumor immune infiltration. Therefore, this study offers insights for further studies on tumor immunotherapy of KIRP and KIRC. The current study is the preliminary part of a larger study, including validation in a study population prospectively enrolled. Undoubtedly, we will make further validation when there are available independent datasets and perform experiments in the future.

## Data Availability Statement

The datasets presented in this study can be found in online repositories. The names of the repository/repositories and accession number(s) can be found in the article.

## Author Contributions

FZ, DS, YX, LJ, and XZ conceived and designed the study. FZ, DS, YX, and LJ performed the analysis procedures. FZ, DS, SC, GW, and YX analyzed the results. YX, SC, KQ, and LJ contributed the analysis tools. FZ, DS, LJ, and XZ contributed to the writing of the manuscript. All authors reviewed the manuscript. All authors contributed to the article and approved the submitted version.

## Funding

This work was supported by the National Natural Science Foundation of China (81770757, 31900902, and 81902603).

## Conflict of Interest

The authors declare that the research was conducted in the absence of any commercial or financial relationships that could be construed as a potential conflict of interest.

## Abbreviations

RCC, Renal Cell Carcinoma; KIRC, Kidney Renal Clear Cell Carcinoma; KIRP, Kidney Renal Papillary Cell Carcinoma; LUAD, Lung Adenocarcinoma; THCA, Thyroid Carcinoma; CESC, Cervical and Endocervical Cancer; CTLA-4, Cytotoxic T - Lymphocyte Antigen 4; LAG-3, Lymphocyte-activation-gene-3; IDO1, Indoximod -1; PD-L2, Programmed death-2; NK cells, Natural Killer cells; NK T cells, Natural Killer T cells; Th 1 cells, Type I helper T cells; Th 2 cells, Type II helper T cells; Treg, Regulatory T cells; Th17 cells, Type 17 T helper cells; Tfh, T follicular helper cell; TAMs, Tumor-Associated Macrophages; TEMs, Tie2-expressing monocytes; TILs, Tumor infiltrating lymphocytes; OS, Overall Survival; RFS, Relapse-Free Survival; DSS, Disease-Specific Survival; DFI, Disease-Free Interval; PFI, Progression-Free Interval; CNV, Copy number variations.
