# Supplementary material for: CTHRC1 Is a Prognostic Biomarker and Correlated With Immune Infiltrates in Kidney Renal Papillary Cell Carcinoma and Kidney Renal Clear Cell Carcinoma
Source: Front Oncol. 2021 Feb 8;10:570819. doi: 10.3389/fonc.2020.570819 (PMC7898899; doi:10.3389/fonc.2020.570819)
Supplement: Supplementary file 1 [file Image_1.pdf]

CTHRC1 is a prognostic biomarker and correlated with immune infiltrates in kidney renal papillary cell carcinoma and kidney renal clear cell carcinoma

Supplementary figures

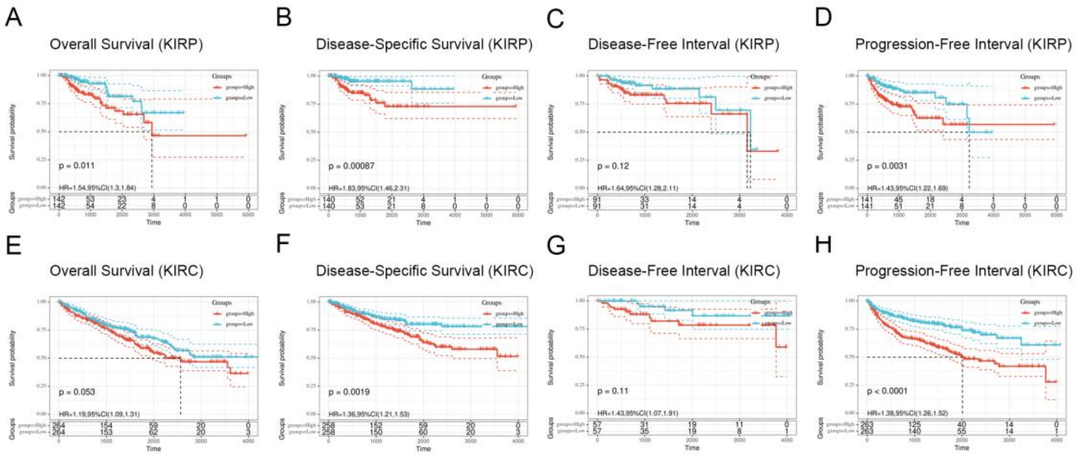

Supplementary Figure S1. Kaplan-Meier survival curve analysis of the prognostic *CTHRC1* up-regulated and down-regulated in KIRP and KIRC. The correlation between *CTHRC1* expression and OS, DSS, DFI, PFI in KIRP cohorts (n=142, n=140, n=90, n=141) **(A-D)**. **(E-H)** Survival curves of OS, DSS, DFI and PFI in KIRC cohort (n=264, n=258, n=57, n=263).

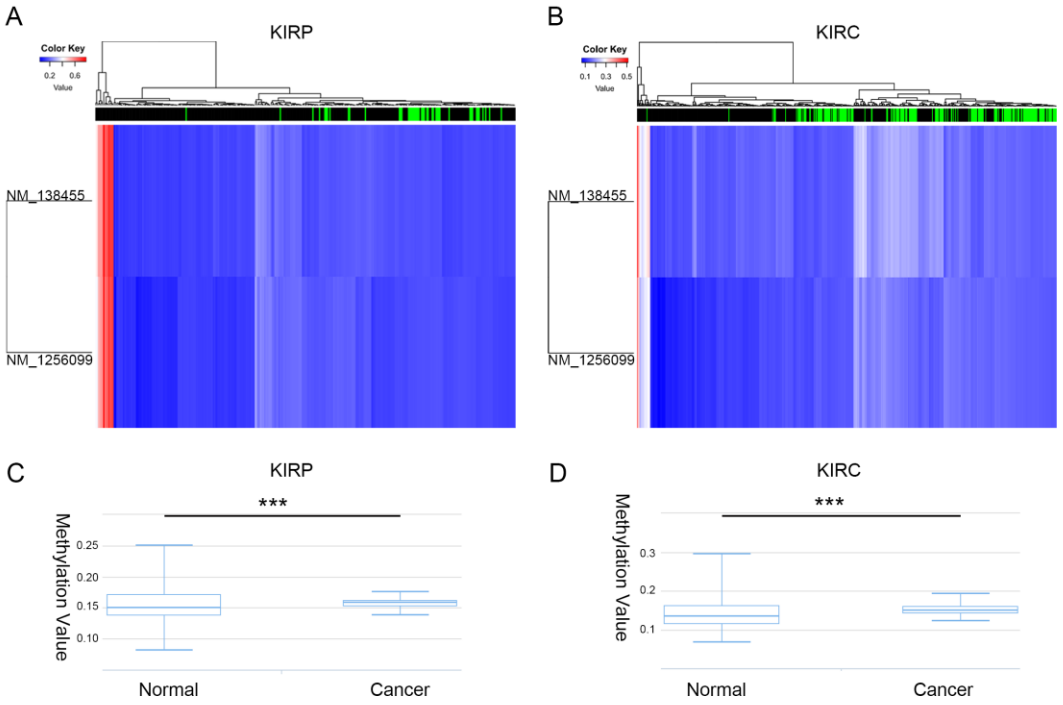

Supplementary Figure S2. DNA methylation analysis of CTHRC1 in DiseaseMeth version 2.0 website. (A) The heatmap reveals methylation of CTHRC1 of 2 transcripts from 321 KIRP samples of 450k. **(B)** The heatmap shows CTHRC1 methylation data of 2 transcripts from 577 KIRC samples of 450k. In the heatmap, rows represent transcripts and columns represent samples (green color represents normal profiles, black represents cancer profiles). **(C-D)** Comparison of CTHRC1 DNA Methylation between normal kidney tissues and KIRP **(C)** and KIRC **(D)** tissues. ***p<0.001.
